# Impact of corticosteroids on the efficacy of CD19/22 CAR-T cell therapy in pediatric patients with B-ALL: a single-center study

**DOI:** 10.3389/fped.2024.1485402

**Published:** 2025-01-13

**Authors:** Jing Yang, Jing Zhang, Xinyu Wan, Jiaoyang Cai, Tianyi Wang, Xiaomin Yang, Wenjie Li, Lixia Ding, Lili Song, Yan Miao, Xiang Wang, Yani Ma, Chengjuan Luo, Jingyan Tang, Longjun Gu, Jing Chen, Jun Lu, Yanjing Tang, Benshang Li

**Affiliations:** ^1^ Department of Cell Immunotherapy, Shanghai Children’s Medical Center, Shanghai Jiao Tong University School of Medicine, Shanghai, China; ^2^ Child Health Advocacy Institute, China Hospital Development Institute, Shanghai Jiao Tong University, Shanghai, China; ^3^ Department of Hematology/Oncology, Children’s Hospital of Soochow University, Suzhou, China

**Keywords:** leukemia, Chimeric antigen receptor, cytokine release syndrome, corticosteroids, children

## Abstract

**Introduction:**

Corticosteroids are used for toxicity management, raising concerns about whether they may affect the anti-leukemic effects of chimeric antigen receptor (CAR)-T cells.

**Methods and results:**

In this study, we retrospectively analyzed patients (fined two subgroups based on disease burden. Of the 75 cases in the low disease burden (LDB) group (MRD < 5%, no extramedullary disease), there was no significant difference between the use of steroids and event-free survival (EFS) (*p* = 0.21) and overall survival (OS) (*p* = 0.26), and the same was found for the 119 cases in the high disease burden (HDB) group. After eliminating the effect of consolidative transplantation on the prognosis, the EFS of the patients who did not use steroids was better (*p* = 0.037) in the LDB group, but the difference was not significant in the HDB group. The median cumulative dexamethasone-equivalent dose was 0.56 mg/kg, and the EFS and OS were similar in the different cumulative dose groups. Furthermore, there was no difference in the recovery of B cells and the expansion of CAR-T cell copies.

**Conclusion and discussion:**

In conclusion, under the guidance of current CRS prevention and control measures, the rational use of corticosteroids does not affect the clinical efficacy and overall survival of CAR-T cell therapy in patients with B-ALL and also does not affect the persistence of CAR-T cells *in vivo*, but the dosage threshold needs further clinical or experimental verification.

## Introduction

Chimeric antigen receptor (CAR)-T cell therapy has yielded remarkable clinical efficacy in relapsed or refractory (r/r) leukemia (1, 2). Our previous phase II clinical study confirmed the safety and efficacy of CD19/22 co-administration (3). However, cytokine release syndrome (CRS) and immune effector cell-associated neurotoxicity syndrome (ICANS) occur with incidence rates of 80%–90% and 20%–72%, respectively, restricting the further promotion of CAR-T cell therapy as a viable treatment option (3–5). CRS management primarily involves anti-cytokine therapy to prevent disease progression or even life-threatening conditions (6–8). The interleukin-6 (IL-6) receptor antagonist tocilizumab is an important treatment for patients with CRS, but it does not cross the blood–brain barrier and tocilizumab may precipitate ICANS in high-risk patients due to the transient increase in the systemic and central nervous system (CNS) IL-6 levels following tocilizumab administration (9, 10). Glucocorticoids (GCs) have a powerful ability to suppress the immune response and are effective in preventing the development of severe CRS or ICANS. GCs have been used in 53% of patients with CRS (11). However, their use can severely impair T-cell function and proliferation (12) and can inhibit CAR-T cell function and induce apoptosis, potentially impairing the effectiveness of CAR-T cell therapy; thus, guidelines do not recommend GCs as a first-line treatment (6, 12, 13). The use of corticosteroids for toxicity management in CAR-T therapy has raised concerns about whether such treatment may affect the anti-leukemic effects of CAR-T cell therapy. Therefore, we conducted a retrospective study to explore whether the use of GCs affects the efficacy of CAR-T cell therapy against leukemia, which will provide a clinical reference for therapy for CAR-T-cell-related toxicities and will contribute to the wider application of CAR-T cell therapy in the treatment of leukemia.

## Methods

This retrospective study included 193 patients aged ≤18 years and 1 younger adult aged 19.6 years who had r/r B-cell acute lymphoblastic leukemia (B-ALL) who were treated at our center with CD19/22 CAR-T cells between September 2019 and December 2021 (Trial Registration No. ChiCTR2000032211, www.chictr.org.cn), as shown in Supplementary Figure S1. We reviewed the medical records of all the enrolled cases and collected information, including grade, duration, and dose of corticosteroids used, and outcome. CRS and ICANS were prospectively evaluated in accordance with the American Society for Transplantation and Cellular Therapy (ASTCT) consensus grading related to immune effector cells, as detailed in Supplementary Tables S1, S2 (13). Medication for CRS and ICANS was administered in accordance with toxicity guidelines for pediatric patients (8).

### Indications for corticosteroid use

According to the ASTCT directives, if the patient is at high risk for severe CRS and ICANS despite the administration of anti-IL-6 medications, it is recommended to administer intravenous dexamethasone at a dosage of 0.5 mg/kg (maximum 10 mg per dose) every 6 h or methylprednisolone at 1–2 mg/kg/day (8). However, based on prior research, corticosteroids may impact CAR-T cell functionality (12, 14), thus, we have established criteria for the administration of low-dose corticosteroids based on clinical experience. We defined a low dose of steroids as the administration of dexamethasone at a temporary dosage of 0.3 mg/kg if the patient's fever remains above 39.5°C despite the use of tocilizumab and antipyretic medications, or alternatively, if norepinephrine has been administered at a rate above 0.3 µg/kg/min, or if the administration of two or more vasoactive agents is required.

### Surveillance of disease condition

#### Minimal residual disease

Minimal residual disease (MRD) refers to the population of leukemia cells that survived treatment and was assessed using an allele-specific quantitative real-time polymerase chain reaction (PCR) and flow cytometry.

#### Complete remission

Complete remission (CR) is defined as no evidence of circulating blasts and no extramedullary disease, including negative cerebrospinal fluid (CSF) tests and <5% bone marrow (BM) blasts.

#### MRD-negative CR

MRD-negative CR is defined as no detectable leukemic cells in the BM, either by PCR or flow cytometry.

#### Event-free survival

Event-free survival (EFS) is defined as the interval between CAR-T cell infusion and the manifestation of any event, encompassing disease progression, recurrence, mortality, secondary tumors, or unacceptable adverse effects. In this study, for patients who underwent a consolidative allogeneic transplantation following CAR-T cell therapy, the EFS duration concluded at the time of the consolidative allogeneic transplantation.

#### Overall survival

Overall survival (OS) is the time from initiation of CAR-T cell infusion until mortality or final follow-up.

#### B-cell recovery

Post-CAR-T infusion, flow cytometry is routinely employed to assess the duration of CD3−/CD19+ B/CD22+ B cell recovery in peripheral blood and bone marrow. B-cell recovery can indirectly assess the durability of functional CAR-T cells *in vivo*.

#### CAR-T cell amplification

CAR-T cell amplification was quantified in patients who volunteered for follow-up by examining peripheral blood samples collected post-CAR-T cell infusion using real-time PCR, with findings expressed as copies per microgram of DNA.

### Criteria for consolidative allogeneic transplantation in patients after CAR-T cell therapy

The criteria for consolidative allogeneic transplantation in patients after CAR-T cell therapy are as follows: patients with CD19/CD22 negative or low CD19/CD22 expression; patients who experienced relapse following two rounds of CAR-T cell therapy; patients with B-cell recovery within 1 month after CAR-T cell infusion; patients with high-risk genetic alterations, including MLL rearrangement, ZNF384 rearrangement, and PAX5 variation; and the patient and/or their guardian have a strong desire for consolidative allogeneic transplantation.

### Statistical methods

Event-free survival and overall survival were estimated using the Kaplan–Meier method and compared using the log-rank test. The association between categorical variables was evaluated using the *χ*^2^ test or Fisher's exact test. The difference in a continuous variable between patient groups was evaluated by the Mann–Whitney test. A *p*-value <0.05 (two-tailed) was considered statistically significant. Statistical analyses were completed using SPSS 24 (IBM) and Prism 9 (GraphPad).

## Results

### Clinical and biological characteristics of patients

Among the 194 patients with relapsed or refractory B-ALL treated with the co-administration of CD19- and CD22-CAR-T cells, the median age was 7.6 years (range 0.8–19.6 years). In total, 75 patients (38.7%) received corticosteroids within 14 days of CAR-T cell infusion, including 1 patient who was improperly administered dexamethasone due to an allergic transfusion response. The clinical and biological characteristics of the 194 individuals were analyzed based on corticosteroid use and are shown in Tables 1, 2.

**Table 1:** Clinical and biological features of the 194 patients with refractory or relapsed B-ALL before CAR-T cell therapy.

Parameter	Total (*n* = 194)	No steroids (*n* = 119)	Steroids (*n* = 75)	*p-*value
Age at infusion (years), median (range)	7.6 (0.8–19.6)	7.1 (0.9–19.6)	7.9 (0.8–17.4)	0.525
Male patient, *n* (%)	128 (66.0)	78 (65.5)	50 (66.7)	0.873
Prior allogeneic transplantation or CD19-CAR-T cell therapy, *n* (%)	14 (7.2)	9 (7.6)	5 (6.7)	0.814
Extramedullary involvement, *n* (%)^a^	48 (24.7)	26 (21.8)	22 (29.3)	0.239
Disease status				
Primary refractory, *n* (%)	22 (11.3)	16 (13.4)	6 (8.0)	0.244
≥1 Relapses, *n* (%)	172 (88.7)	103 (86.6)	69 (92.0)	
MRD prior to CAR-T cell therapy (%)				
<5	108 (55.7)	73 (61.3)	35 (46.7)	0.045*
≥5	86 (44.3)	46 (38.7)	40 (53.3)	
High-risk cytogenetics^b^	56 (28.9)	38 (31.9)	18 (24.0)	0.235
Total CAR-T cell infusion dose (E6/kg)	5.6 [1.3–13.0]	6.0 [1.3–13.0]	5.4 [1.7–12.5]	0.884
Disease burden prior to CAR-T cell therapy^c^				
Low disease burden, *n* (%)	75 (38.7)	52 (43.7)	23 (30.7)	0.070
High disease burden, *n* (%)	119 (61.3)	67 (56.3)	52 (69.3)	

MRD, minimal residual disease; BCR-ABL1, Fusion gene formed by a break and translocation of the chromosomes 9 and 22; TCF-HLF, Fusion gene formed by chromosome translocation t(17; 19) (q22; p13); KMT2A rearrangement, lysine methyltransferase 2A, located on chromosome 11q23; ZNF384, zinc finger protein 384, located on chromosome 12p13; MEF2D-rearrangement, myocyte enhancer factor 2D, located on chromosome1q22; iAMP21, intrachromosomal amplification of chromosome 21.

0.045*: there was a significant difference in MRD between the steroids group and the non-steroids group, so we stratified the patients according to disease burden. Disease burden prior to CAR-T cell therapyc: High disease burden: MRD ≥ 5%, with CNS involvement, and/or with non-CNS extramedullary disease. Low disease burden: MRD < 5%, absence of CNS involvement, and no other extramedullary disease.

^a^ Extramedullary involvement: sites include CNS (*n* = 26), testes (*n* = 14), CNS and testes (*n* = 5), kidneys (*n* = 2), and bones (*n* = 1).

^b^ High-risk cytogenetics: BCR–ABL1, TCF–HLF, KMT2A rearrangement, ZNF384, MEF2D-rearrangement, iAMP21.

^c^ Low disease burden: MRD < 5%, absence of CNS involvement, and no other extramedullary disease.

*p < 0.05, the results were statistically significant.

**Table 2:** Clinical and biological features of the 194 patients with refractory or relapsed B-ALL after CAR-T cell therapy.

Parameter	Total (*n* = 194)	No steroids (*n* = 119)	Steroids (*n* = 75)	*p-*value
After receiving CD19/CD22 CAR-T cell therapy				
IL-6, median (range)	1,371.8 (1.10–43,898.9)	386.7 (1.10–17,307.3)	7,768.3 (3.00–43,898.9)	<0.001*
IFN-γ, median (range)	223.6 (0.00–20,455.4)	36.9 (0.00–16,944.9)	1,174.9 (0.85–20,455.4)	<0.001*
CRS grade any, *n* (%)	171 (88.1)	96 (80.7)	75 (100)	<0.001*
1	74 (38.1)	64 (53.7)	10 (13.3)	
2	43 (22.2)	21 (17.6)	22 (29.3)	
3	38 (19.6)	11 (9.2)	27 (36)	
4	16 (8.2)	0 (0)	16 (21.3)	
Time to onset of CRS (days), median (range)	1 (0–10)	2 (0–10)	1 (0–5)	<0.001*
Duration of CRS (days), median (range)	5 (1–18)	4 (1–18)	5 (1–14)	<0.001*
ICANS (≥grade 2), *n* (%)	21 (10.82)	3 (2.52)	18 (24)	<0.001*
Consolidative transplantation after CD19/22-CAR-T cell therapy, *n* (%)	78 (40.2)	41 (34.5)	37 (49.3)	0.040*

CRS, cytokine release syndrome; ICANS, immune effector cell-associated neurotoxicity syndrome.

0.040*: There was a significant difference in Consolidative transplantation between the steroids group and the non-steroids group, so we stratified the patients according to it.

*p < 0.05, the results were statistically significant.

The patients administered with corticosteroids exhibited a higher MRD rate (5.95% vs. 2.31%, *p* = 0.005), likely due to an increased tumor burden resulting in enhanced antigen stimulation, which subsequently induces a more pronounced CRS response. Consequently, the corticosteroid-treated patients showed elevated peaks of IL-6 (7,768 vs. 386 pg/ml, *p* < 0.001) and interferon-gamma (IFN-γ) following CAR-T cell therapy (1,174.9 vs. 36.9 pg/ml, *p* < 0.001) (Supplementary Figure S2). We established two subgroups based on disease burden: the low disease burden (LDB) group, characterized by an MRD <5%, the absence of CNS involvement, and no other extramedullary disease; and the high disease burden (HDB) group defined by an MRD ≥5%, with CNS involvement, and/or with non-CNS extramedullary disease.

CRS was found in 171 individuals (88%), with 54 patients (28%) experiencing grade ≥3 CRS. Furthermore, 21 patients (10.8%) experienced two or more instances of ICANS grading (Table 2). In comparison with patients in the non-steroid group, individuals in the steroid group experienced an earlier onset and longer duration of CRS (Table 2). The median start of CRS occurred on day 1 (range of 0–5 days), and the median duration of CRS was 5 days (range of 1–18 days) following CAR-T cell treatment. The effectiveness of the treatment was evaluated in all 194 patients, with 192 (99%) achieving a negative MRD status. The median follow-up duration was 14 months post-infusion (interquartile range of 9–21 months). We analyzed the 24-month EFS and OS between patients who received steroids and those who did not, based on various factors (Table 3). The findings in this section demonstrate that there was no significant difference in survival rates between the two groups when considering disease state and MRD classification. Patients with grade 1 CRS had worse EFS (*p* = 0.024) in the steroid group compared to the non-steroid group (Supplementary Figure S3). Patients with ≥grade 2 ICANS who took steroids had a superior overall survival compared to those who did not (*p* = 0.014).

**Table 3:** Comparison of EFS and OS between the 75 patients corticosteroids used and the 119 individuals non-steroids based on various characteristics.

Parameter	Total, *n* (%)	No steroids (*n* = 119)			Steroids (*n* = 75)			*p-*value (EFS) from log-rank test	*p-*value (OS) from log-rank test
		Total, *n* (%)	24-month EFS (95% CI)	24-month OS (95% CI)	Total, *n* (%)	24-month EFS (95% CI)	24-month OS (95% CI)		
Disease status									
Primary refractory	22 (11.3)	16 (13.4)	64 (34.6–93.4)	87 (69.4–100)	6 (8.0)	71 (24.0–100)	100	0.89	0.38
First relapse	136 (70.1)	87 (73.1)	62 (46.3–77.7)	83 (73.2–92.8)	49 (65.3)	77 (63.3–90.7)	93 (85.2–100)	0.82	0.29
≥2 relapses	36 (18.6)	16 (13.4)	54 (18.7–89.3)	63 (33.6–92.4)	20 (26.7)	65^a^ (33.6–96.3)	75 (55.4–94.6)	0.88	0.99
MRD before CAR-T									
<5%	108 (55.7)	73 (61.3)	71 (57.3–84.7)	84 (74.2–93.8)	35 (46.7)	81^b^ (63.4–98.7)	88 (78.2–97.8)	0.82	0.78
≥5%	86 (44.3)	46 (38.7)	46 (22.5–69.5)	77 (63.3–90.7)	40 (53.3)	69 (51.4–86.6)	89 (79.2–98.8)	0.94	0.22
CRS grading									
1	74 (38.1)	64 (53.7)	78 (64.3–91.7)	80 (68.2–91.8)	10 (13.3)	52^c^ (0–100)	90 (72.4–100)	**0**.024*	0.67
2	43 (22.2)	21 (17.6)	41 (1.8–80.2)	95 (85.2–100)	22 (29.3)	84 (64.4–100)	89 (75.3–100)	0.32	0.65
3	38 (19.6)	11 (9.2)	37^d^ (0–74.2)	68 (38.6–97.4)	27 (36)	74 (52.4–95.6)	89 (77.2–100)	0.11	0.22
4	16 (8.2)	0 (0)	/	/	16 (21.3)	48^e^ (4.9–91.1)	86 (68.4–100)	/	/
ICANS ≥ grade 2									
Yes	21 (10.82)	3 (2.5)	50^c^ (0–100)	33^f^ (0–85.9)	18 (24)	65 (35–100)	89 (73.3–100)	0.104	**0**.014*
No	173 (89.2)	116 (97.5)	62 (48.3–75.7)	83 (75.2–90.8)	57 (76)	73 (15.7–88.7)	88 (78.2–97.8)	0.78	0.84

MRD, minimal residual disease; CRS, cytokine release syndrome; ICANS, immune effector cell-associated neurotoxicity syndrome; EFS, event-free survival; OS, overall survival.

0.024*: among patients who developed grade 1 CRS, those who did not receive corticosteroids exhibited superior EFS.

0.014*: among patients with ICANS ≥ grade 2, the steroids group exhibited superior OS.

^a^ 18-month EFS.

^b^ 20-month EFS.

^c^ 3-month EFS.

^d^ 12-month EFS.

^e^ 6-month EFS.

^f^ 18-month OS.

*p < 0.05, the results were statistically significant.

### Outcomes depending on disease burden

**Figure 1:**
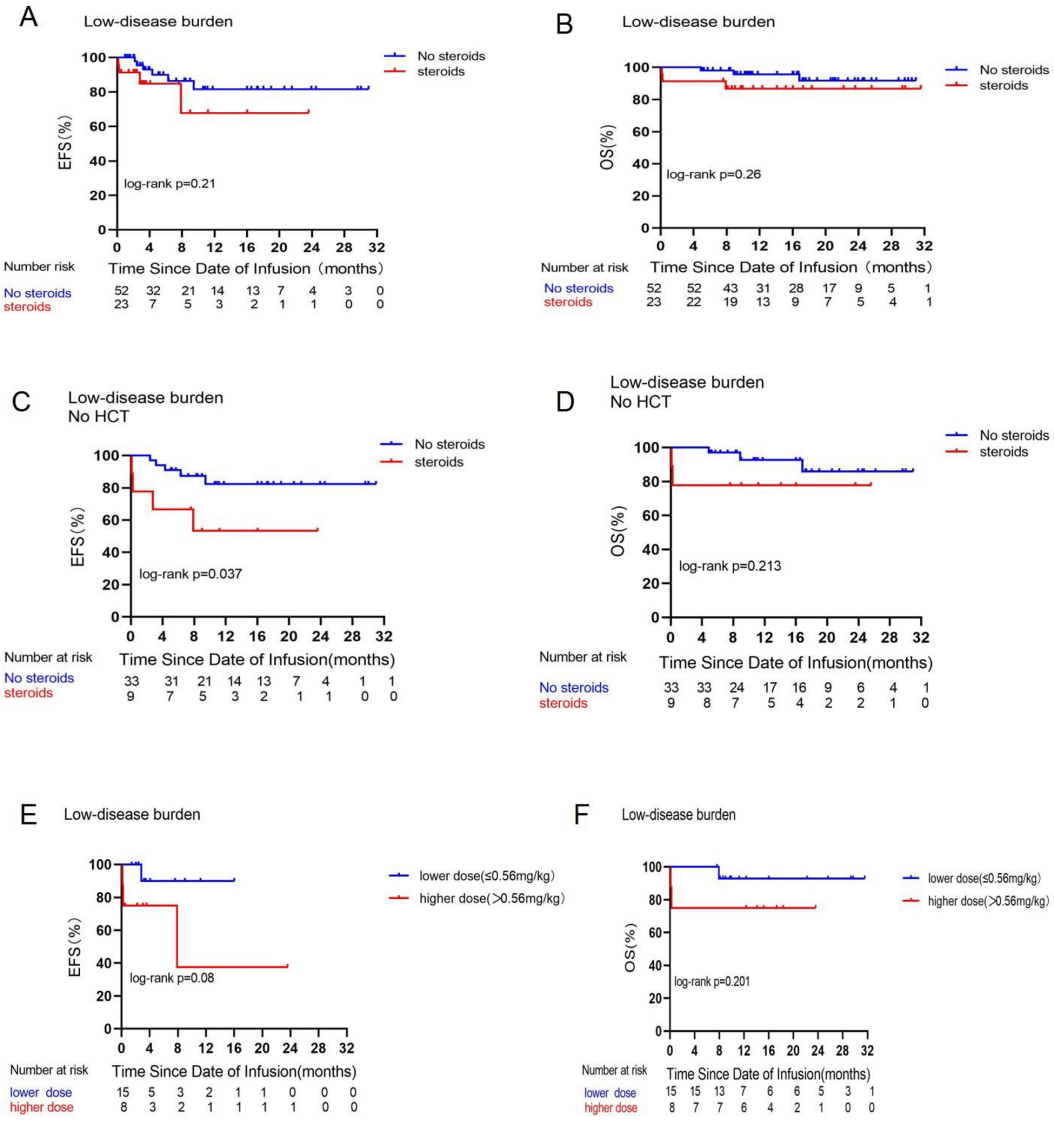
Prognostic impact of corticosteroid use on EFS and OS in the low disease burden group. Comparisons of EFS **(A)** and OS **(B)** between patients who did or did not receive corticosteroids in the low disease burden group. Comparative analysis of EFS **(C)** and OS **(D)** among patients in the low disease burden group who either received or did not receive corticosteroids and did not undergo consolidative allogeneic transplantation following CAR-T cell treatment. Comparisons of EFS **(E)** and OS (**(F)** between patients who received a low dose (≤0.56 mg/kg) or high dose (>0.56 mg/kg) of dexamethasone in the low disease burden group.

**Figure 2:**
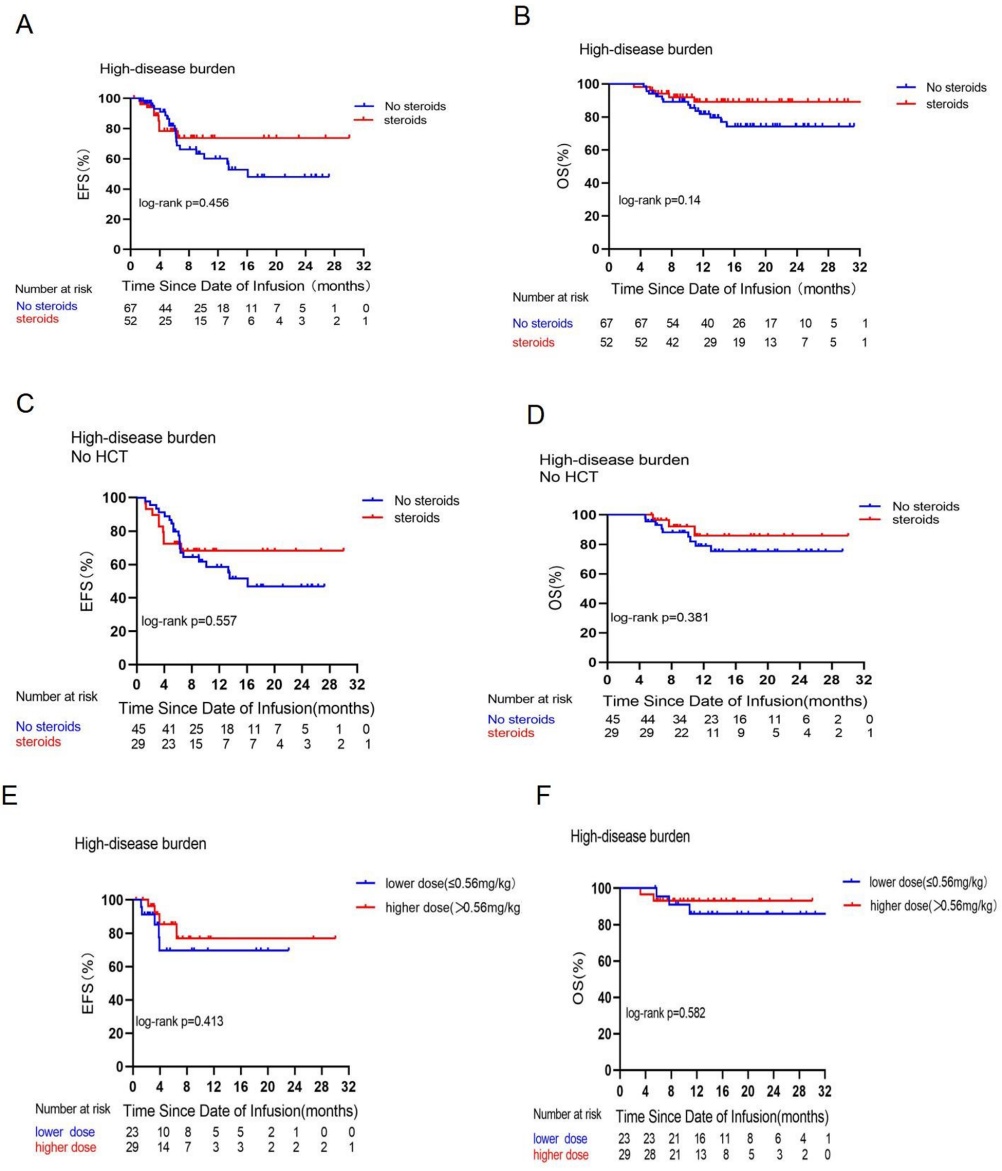
Prognostic impact of corticosteroid use on EFS and OS in the high disease burden group. Comparisons of EFS **(A)** and OS **(B)** between patients who did or did not receive corticosteroids in the high disease burden group. Comparisons of EFS **(C)** and OS **(D)** between patients who did or did not receive corticosteroids in the high disease burden group and did not receive consolidative allogeneic transplantation [No hematopoieticstem cell transplantation (HCT] after CAR-T cell therapy. Comparisons of EFS **(E)** and OS **(F)** between patients who received a low dose (≤0.56 mg/kg) or a high dose (>0.56 mg/kg) of dexamethasone in the high disease burden group.

This retrospective analysis assessed the effectiveness of treatment in 194 patients. The MRD-negative complete responses were 119/119 (100%) in the non-steroid cohort and 73/75 (97.3%) in the steroid cohort (*p* = 0.073). Based on disease burden, there were 75 cases in the LDB group and 119 instances in the HDB group, with 23 and 52 patients receiving corticosteroids in each group respectively. No significant changes in EFS and OS were found due to corticosteroid treatment in either the LDB (Figures 1A,B) or HDB (Figures 2A,B) groups. To mitigate the impact of consolidative transplantation on the prognosis, we identified 116 patients who did not undergo consolidative allogeneic transplantation following CAR-T cell treatment (Figures 1C,D, 2C,D). Our findings indicated that corticosteroid usage was associated with reduced EFS (*p* = 0.037) in the LDB group (Figure 1C). Nonetheless, this outcome was not observed in the HDB group, where 74 individuals exhibited analogous survival curves (Figures 2C,D) contingent upon corticosteroid usage. We also examined the correlation between corticosteroid dosage and prognosis. The median cumulative dosage of intravenous dexamethasone was 0.56 mg/kg (range of 0.04–13.87 mg/kg), based on which we categorized the subjects into two groups. Among the 75 individuals administered with corticosteroids, no statistically significant difference (Figures 2E,F) was seen in EFS (*p* = 0.413) and OS (*p* = 0.582) within the HDB group. The use of reduced cumulative dexamethasone indicated a tendency toward increased EFS (*p* = 0.08) in the LDB group (Figure 1E). Furthermore, 77 patients (64.7%) in the HDB group developed grade 0–2 CRS, whereas 42 patients (35.3%) experienced severe grade 3 or 4 CRS (Supplementary Figure S4). In instances of grade 3 or 4 CRS, as seen in Supplementary Figures S4C,D, EFS (*p* = 0.034) and OS (*p* = 0.062) were higher in the steroid group compared with the non-steroid group.

**Figure 3:**
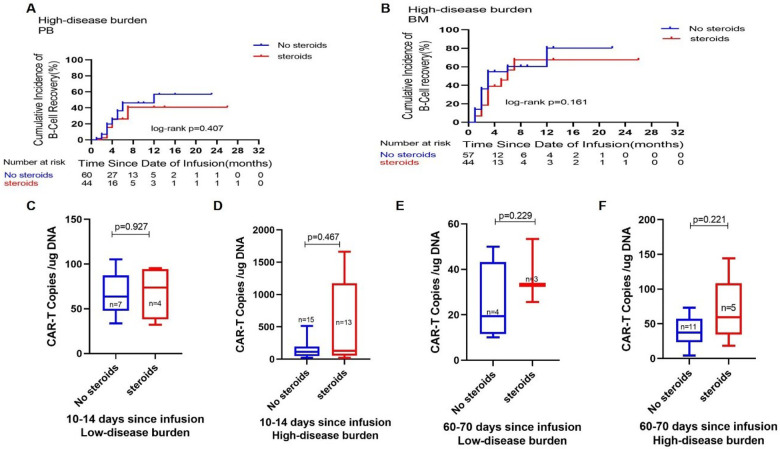
Impact of corticosteroid use on CAR-T cell metabolism. Comparisons of the cumulative incidence of B-cell recovery in the peripheral blood **(A)** and bone marrow **(B)** between patients who did or did not receive corticosteroids in the high disease burden group. Comparison of CAR-T cell copies on days 10–14 **(C)** and days 60–70 **(E)** after CAR-T cell infusion between patients who did or did not receive corticosteroids in the low disease burden group. Comparison of CAR-T cell copies on days 10–14 **(D)** and days 60–70 **(F)** after CAR-T cell infusion between patients who did or did not receive corticosteroids in the high disease burden group.

Subsequently, we examined the correlations between B-cell reconstruction and CAR-T cell quantities in connection with corticosteroid administration. In the HDB group, Figure 3 illustrates the comparative B-cell reconstruction curves for the steroid and non-steroid groups, revealing no statistically significant changes in peripheral blood (Figure 3A) and bone marrow (Figure 3B) between the two cohorts. We observed CAR-T cell copy numbers in certain individuals following CAR-T cell infusion, as seen in Figures 3C–F. In both the LDB and HDB groups, there were no statistically significant changes in the proliferation of CAR-T cell copies at 10–14 or 60–70 days post-infusion in relation to corticosteroid administration.

## Discussion

The treatment of toxicity in CAR-T therapy is an emerging topic, with ongoing research aimed at enhancing safety while preserving the longevity of the therapeutic benefit. This retrospective investigation posits that steroids do not affect the remission rate of CAR-T cell treatment in leukemia, consistent with the findings of Liu et al. (15). Low-dose corticosteroids were employed as first-line treatment to manage CRS in patients with B-ALL undergoing CD19- or CD22-CAR-T cell therapy, encompassing both children and adults; however, a comparison of survival between the two groups was not conducted.

This study, by categorizing varying disease burdens as seen in Figures 1B, 2B, indicates that corticosteroid administration did not influence the OS of patients undergoing CAR-T cell treatment for leukemia. This contrasts with the findings of Strati et al. (16), who retrospectively analyzed steroid usage in 100 adults with large B-cell lymphoma (LBCL) undergoing CAR-T cell therapy, with 60 subjects utilizing corticosteroids while 40 did not, and concluded that steroid use significantly diminished overall survival. The discrepancies in results may be attributed to the following factors. First, an examination of the research participants reveals that our study focuses on pediatric patients with B-ALL, all under the age of 20, whereas the international research subjects consisted of adults with LBCL. Research indicates that the process by which CAR-T cells eliminate tumor cells is primarily governed by three axes: the perforin and granzyme axis, cytokine secretion, and the Fas and FasL axis (17, 18). Larson et al. (19) discovered that solid tumors and liquid tumors exhibit distinct interaction mechanisms with CAR-T cells, indicating that solid tumors with defects in the IFN-γ receptor gene demonstrate greater resistance to CAR-T cell cytotoxicity, whereas liquid tumors are not rendered insensitive to CAR-T cells due to IFN-*γ* receptor gene defects. One mechanism by which GCs suppress the immune response is through the inhibition of T-cell activity by reducing cytokine production, including IFN-γ (12). We hypothesize that this may be attributed to the differing interactions of CAR-T cells with solid tumors vs. leukemia; CAR-T cells that target solid tumors depend on the IFN-γ receptor signaling pathway for cytotoxicity, whereas leukemia cells exhibit reduced reliance on this pathway. The use of modest dosages of corticosteroids did not impact the overall survival of leukemia patients; nevertheless, this conclusion requires to be validated via further studies.

Conversely, our center adheres to stringent standards regarding the indication for corticosteroid use, as outlined in the Methods section. Consequently, only 44% of patients with CRS utilized corticosteroids, with a median cumulative dexamethasone-equivalent dosage of 0.56 mg/kg; the majority were administered a single dose, and corticosteroids were promptly withdrawn upon improvement of the clinical symptoms. Only three individuals experienced persistent grade 4 CRS due to the ongoing use of high-dose methylprednisolone.

An *in vitro* study by Brummer et al. (20) that treated glioblastoma cell lines with CAR-T cells and dexamethasone demonstrated the efficacy of high concentrations of dexamethasone to antagonize CAR-T cells by depleting or reducing the activity of CAR-T cells and promoting tumor cell growth. The cumulative dosage of dexamethasone exceeding a threshold level may influence CAR-T cell functionality. In a mouse validation experiment (21), researchers discovered that high dosages of dexamethasone (>5 mg/kg) abolished CAR-T-cell-mediated tumor eradication, but CAR-T cell tumor lysis was sustained at lower doses of 0.2 or 1 mg/kg of dexamethasone. Our investigation indicates that, as seen in Figures 1E,F, elevated dosages of dexamethasone (more than 0.56 mg/kg) impaired EFS and OS in the LDB group; nevertheless, the difference lacks statistical significance. Furthermore, only three individuals received a cumulative dose of dexamethasone greater than 5 mg/kg. Consequently, we hypothesize that the administration of dexamethasone in the patients in this trial may not have attained the dosage required to influence CAR-T cell impairment.

The timing of corticosteroid administration in the treatment of clinical toxicity remains a contentious topic. Strati et al. (16) posited that corticosteroid administration should be postponed as long as the CRS response is manageable. Some research studies suggest that early corticosteroid intervention may mitigate the risk of severe CRS and ICANS, benefiting patients without compromising therapeutic effectiveness (15, 22, 23). We observed that individuals who were administered corticosteroids exhibited more favorable outcomes during severe grade 3 or 4 CRS in the HDB group (Supplementary Figure S4C). The HDB group also included nine patients who did not receive corticosteroids and the biological and clinical characteristics of this group are detailed in Supplementary Table S3. Three patients had a risk gene (MLL rearrangement positive). We propose that this may be a contributing factor to the worse prognosis.

Disease recurrence after remission with CAR-T cell therapy remains challenging, with several studies indicating that the depletion of CAR-T cells is a primary factor contributing to tumor recurrence (24). The use of corticosteroids following CAR-T cell infusion in certain individuals did not influence the growth or durability of CAR-T cells (Figure 3). The quantity of CAR-T cells in the steroid group was marginally higher than in the non-steroid group, potentially attributable to their elevated tumor burden. The increased antigen stimulation from CAR-T cell infusion may lead to a significant increase in CAR-T cells, resulting in a more pronounced CRS response, thereby increasing the likelihood of corticosteroid administration.

Greater tumor burden was linked to an elevated likelihood of severe CRS (25). In our LDB group, those who did not receive corticosteroids or were administered lower doses (Figures 1C,E) exhibited superior EFS. However, Oluwole et al. (26) administered dexamethasone prophylactically to LBCL patients on the day prior to CAR-T cell infusion and they discovered that preventive and early corticosteroids treatment resulted in no grade 3 or higher CRS but had a significant response rate. Consequently, corticosteroid utilization can be reduced when the disease burden is low; conversely, a high disease burden increases the risk of grade 3 or 4 CRS reactions. Early administration of corticosteroids may facilitate prompt management of life-threatening CRS reactions, enabling immediate cessation of the treatment once the reaction is clinically manageable.

### Limitations

This is a retrospective study with a small sample size, and the CAR-T cell products utilized are not yet commercially available, potentially resulting in selection bias in the study outcomes. This study is now an ongoing Phase II clinical trial, and we are enlisting additional patients and reassessing patient data to further validate our findings and derive further conclusions.

## Conclusion

These data suggest that the judicious application of corticosteroids, in accordance with existing CRS prevention and control protocols, may not influence the clinical effectiveness or overall survival of r/r B-ALL patients undergoing CAR-T treatment. In CAR-T cell therapy for leukemia patients, a clinician should avoid administering corticosteroids when the disease burden is low. Conversely, when the disease burden is high, the risk of grade 3 or 4 CRS reactions increases, and corticosteroids may be administered early to prevent life-threatening CRS reactions until the clinical symptoms improve, after which they should be discontinued immediately.

## Data Availability

The raw data supporting the conclusions of this article will be made available by the authors, without undue reservation.
