# Supplementary material for: Impact of corticosteroids on the efficacy of CD19/22 CAR-T cell therapy in pediatric patients with B-ALL: a single-center study
Source: Front Pediatr. 2025 Jan 13;12:1485402. doi: 10.3389/fped.2024.1485402 (PMC11771322; doi:10.3389/fped.2024.1485402)
Supplement: Supplementary file 1 [file Datasheet1.docx]

Supplementary Material

**Supplementary Figure 1.**

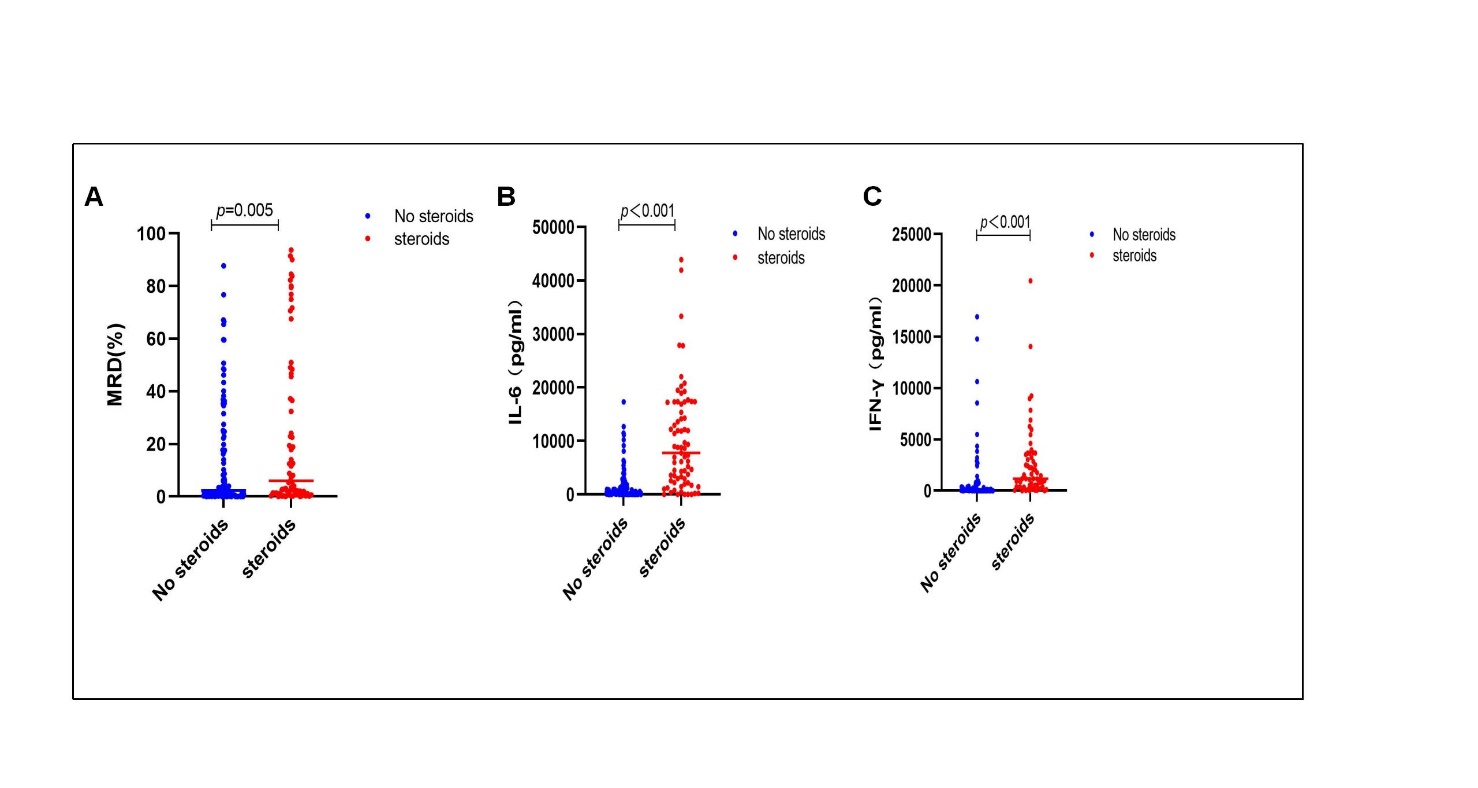
**Supplementary Figure 2.**

**Supplementary Figure 2.** Comparison of MRD(A), peak levels of interleukin 6 (IL-6) (B), and interferon-gamma (IFNγ) (C) based on corticosteroid use. The MRD prior to CAR-T therapy, as well as peak concentrations of IL-6 and INFγ following CAR-T, were markedly elevated in patients receiving steroids compared to those who did not utilize steroids.

**Supplementary Figure 3.**


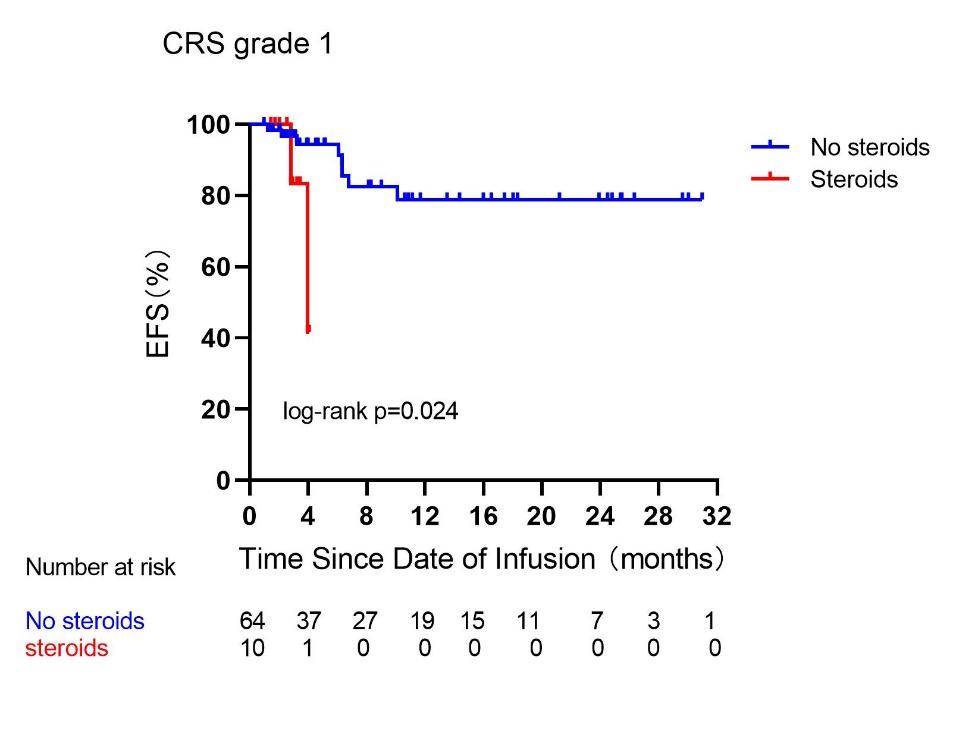


**Supplementary Figure 3.** Comparison of EFS in patients with CRS grade 1 based on corticosteroids administration.

**Su****pplementary Figure 4.**

**.
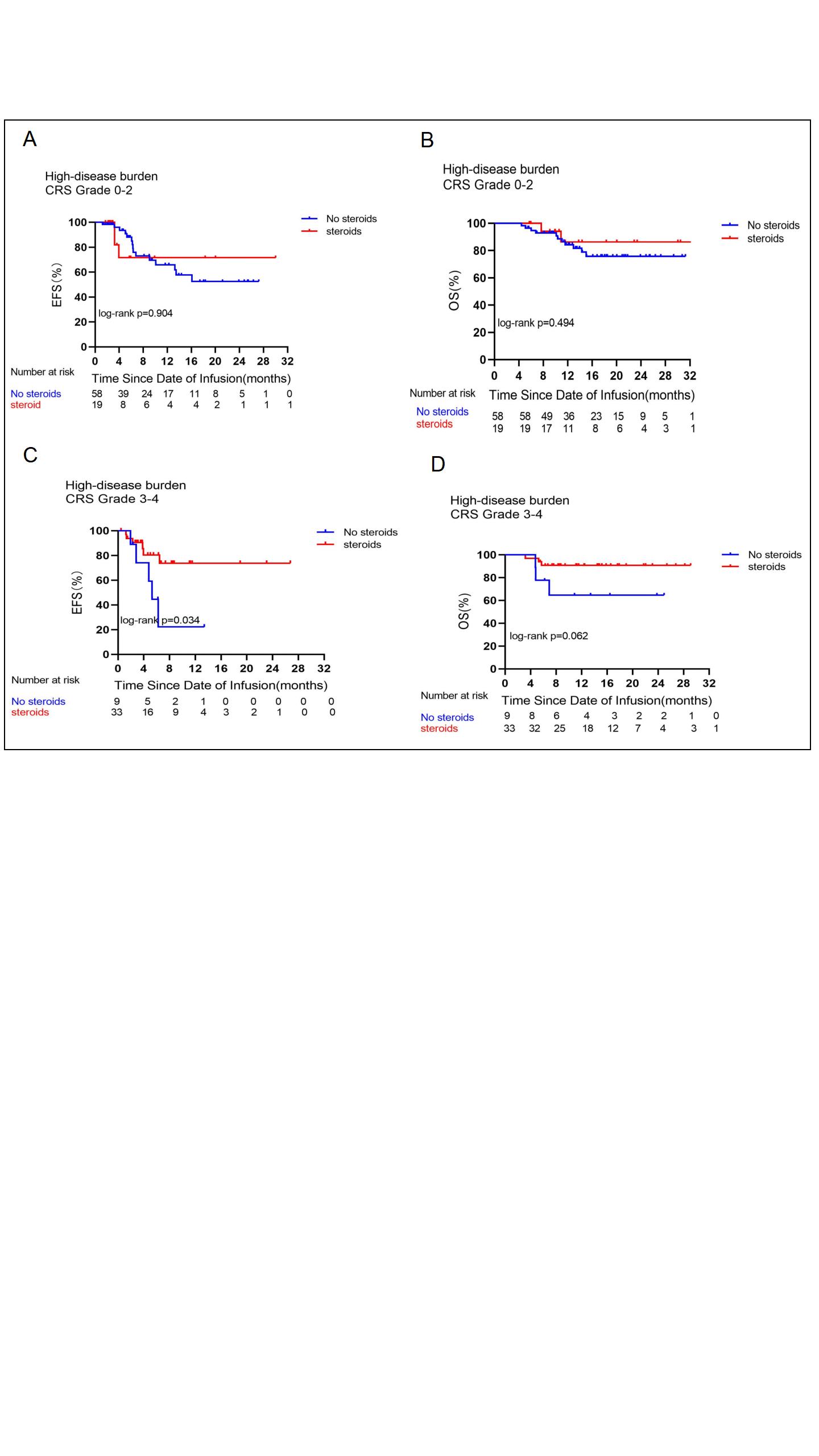
**

**Supplementary F****igure 4. Prognostic impact of corticosteroids use on EFS and OS in the high-disease burden group based on CRS grading.** Comparisons of EFS(A) and OS(B) between patients with CRS Grade 0-2 who did or did not receive corticosteroids in the high-disease burden group. Comparisons of EFS(C) and OS(D) between patients with CRS Grade 3-4 who did or did not receive corticosteroids in the high-disease burden group.

**Supplementary Table1**. ASTCT CRS Consensus Grading

| **CRS Parameter** | **Grade 1** | **Grade 2** | **Grade 3** | **Grade 4** |
| --- | --- | --- | --- | --- |
| **Fever** | Temperature ≥38°C | Temperature ≥38°C | Temperature ≥38°C | Temperature ≥38°C |
|  | | With | | |
| **Hypotension** | None | Not requiring vasopressors | Requiring a vasopressor with or without vasopressin | Requiring multiple vasopressors (excluding vasopressin) |
|  | | And/or | | |
| **Hypoxia** | None | Requiring low-flow nasal cannula or blow-by | Requiring high-flow nasal cannula, facemask, nonrebreather mask, or Venturi mask | Requiring positive pressure (eg, CPAP, BiPAP, intubation and mechanical ventilation) |

**Supplementary Table2.** ASTCT ICANS Consensus Grading

| **ICANS Parameter** | **Grade 1** | **Grade 2** | **Grade 3** | **Grade 4** |
| --- | --- | --- | --- | --- |
| **ICE/CAPD score*** | 7-9/1-8 | 3-6/1-8 | 0-2/≥9 | 0 (patient is unarousable and unable to perform ICE/CPAD) |
| **Depressed level of consciousness** | Awakens  spontaneously | Awakens to voice | Awakens only to tactile stimulus | Unarousable or requires vigorous or repetitive tactile stimuli to arouse; stupor or coma |
| **Seizure** | N/A | N/A | Any clinical seizure focal or generalized that resolves rapidly or nonconvulsive seizures on EEG that resolve with intervention | Life-threatening prolonged seizure (>5 min); or Repetitive clinical or electrical seizures without return to baseline in between |
| **Motor findings** | **N/A** | N/A | N/A | Deep focal motor weakness such as hemiparesis or paraparesis |
| **Elevated ICP/ cerebral edema** | N/A | N/A | Focal/local edema on neuroimaging | Decerebrate or decorticate posturing, cranial nerve VI palsy, papilledema, Cushing's triad, or signs of diffuse cerebral edema on neuroimaging |

* ICANS grade is determined by the most severe event ICE score(≥12 years old) or CAPD score(＜12 years old).

ICE Score for patients age ≥12 years old (Orientation 4 points; Naming 3 points; Following commands 1 point; Writing 1point; Attention 1point)

CAPD score for patients age ＜12 years old（Observe the children's condition including Mental state and general situation, like eye contact, action, communicate needs et al.

**Supplementary Table 3.** Biological and clinical features of 9 patients in Supplementary Figure 4C.

|  | | | | | | | | | |
| --- | --- | --- | --- | --- | --- | --- | --- | --- | --- |
| Patient No. | | Age | Sex | Genotype | Relapse site | Event | EFS | BMT after CAR-T | B-Cell recovery in 3months |
| 1 | 13.4 | | Male | ASXL1 | BM | / | 2.3 | Yes | No |
| 2 | 7.6 | | Male | ETV6-RUNX1 | BM, Testis | Relapse | 6.2 | No | No |
| 3 | 6.2 | | Male | Negative | BM | Relapse | 1.9 | No | No |
| 4 | 12.9 | | Female | **KMT2A-MLLT1** | BM | Relapse | 5.3 | No | Yes |
| 5 | 1.2 | | Male | **KMT2A-EPS15** | BM | Relapse | 2.8 | No | Yes |
| 6 | 7.9 | | Male | NRAS:G13D: CREBBP:D1435V | BM, CNS, Testis | / | 13.4 | No | No |
| 7 | 9.9 | | Male | IKZF1-IK6 | BM | / | 2.3 | Yes | No |
| 8 | 8.2 | | Female | Negative | BM, CNS | / | 6.2 | No | No |
| 9 | 1.4 | | Male | **KMT2A-MLLT1** | BM, CNS | Death of viral encephalitis | 4.8 | No | No |
